# *Kadsura coccinea* Lignan Metabolism Based on Metabolome and Transcriptome Analysis

**DOI:** 10.1155/2022/3152155

**Published:** 2022-08-01

**Authors:** Zhonghou Liang, Xiang Li, Ping Li, Ye Deng, Yuan Zhong, Hua Yang

**Affiliations:** ^1^Hunan Engineering Research Center for Applied Technology of Understory Medicinal Plants, Hengyang 421005, China; ^2^Hunan Polytechnic of Environment and Biology, Hengyang 421005, China; ^3^Qidi Guhan Group Hengyang Traditional Chinese Medicine Co., Ltd, Hengyang 421005, China; ^4^College of Bioscience and Biotechnology, Hunan Agricultural University, Changsha 410128, China

## Abstract

*Kadsura coccinea* is an important resource of traditional Chinese medicine. We find out the gene information of enzymes related to lignan biosynthesis and metabolism of *Kadsura coccinea*, so as to provide a scientific basis for the breeding of new varieties of *Kadsura coccinea*. In this paper, 2-year-old *Kadsura coccinea* from Hunan *Kadsura coccinea* provincial germplasm resource bank was used as the material and its root, stem, and leaf were analyzed by extensive targeted metabolomics combined with transcriptome sequencing. The results showed the following: (1) 51 lignans were detected by metabolome analysis, and the content of lignans in roots was higher than that in stems and leaves. The high content of lignans in roots, stems, and leaves includes ring-opening isolarch phenol-4-o-glucoside, narrow leaf schisandrin E, and schisandrin B. (2) After transcriptome sequencing, 13 classes of 137 Unigenes related to lignan biosynthesis pathway were retrieved. The analysis of differential genes in different parts showed that the overall expression amount and species of *Kadsura coccinea* lignan synthase gene in stems and leaves were closer than those in roots. CCoAOMT, C3H, and SIDR gene families are mainly expressed in roots and stems. (3) Metabolome combined with transcriptome analysis further screened these genes and obtained 11 genes of enzyme gene families such as HCT, DIR, COMT, CAD, SIDR, and PLR, which are highly correlated in lignan synthesis. Therefore, there are many lignans and their synthase-related genes in *Kadsura coccinea* roots, stems, and leaves, but the content and expression of different lignans and their synthase-related genes are quite different in each part. In this study, the gene information of the *Kadsura coccinea* lignan biosynthesis enzyme was obtained for the first time, which laid a good foundation for the cloning and molecular breeding of the key enzyme gene of lignan biosynthesis.

## 1. Introduction

*Kadsura coccinea* is an evergreen woody vine of the *Schisandra* genus in the Schisandra family, also known as cold rice ball, bufuna, chicken intestine wind, etc. *Kadsura coccinea* is an important medicinal resource, mainly distributed in Hunan, Guangxi, Guizhou, Fujian, and other places [1]. Folk medicine mostly uses its roots, vines, and fruits to treat stomach diseases, rheumatoid arthritis, bruises, swelling and pain, irregular menstruation, and so on [2]. Pharmacological studies showed that *Kadsura coccinea* has the effects of antitumor [1], antioxidation [3], liver protection [4], and inhibition of acetylcholinesterase [5].

The main active components of *Kadsura coccinea* are lignans, triterpenes, sesquiterpenes, sterols, and other compounds, among which lignans are the main active components [6]. At present, 121 kinds of lignans have been isolated and identified, mainly including biphenyl cyclooctadiene type, spirobenzofuran type, and 6, 9-oxbridge biphenyl cyclooctadiene type, followed by a small amount of arylnaphthalene type and dibenzylbutane type lignans [7]. Guo Xia et al. [8] measured the highest content of total lignans in Huaihua, Hunan Province, from *Kadsura coccinea* varieties collected from six different provenances. At present, the research on *Kadsura coccinea* mainly focuses on chemical composition analysis, pharmacological activity screening, and cultivation [9]. However, the analysis of the *Kadsura coccinea* secondary metabolic pathway has not been carried out, and the discovery of key enzyme genes is also rare. With the development of gene sequencing, based on extensive targeted metabolome combined with transcriptome sequencing analysis, studying the biosynthetic regulation pathway of *Kadsura coccinea* lignans will contribute to the development of molecular markers of *Kadsura coccinea*, carry out molecular breeding, cultivate excellent *Kadsura coccinea* varieties with high lignan content, and lay a foundation for the further development and utilization of *Kadsura coccinea* traditional Chinese medicine resources.

## 2. Materials and Methods

### 2.1. Materials

The experimental material *Kadsura coccinea* comes from the provincial Germplasm Resource Bank of *Kadsura coccinea* in Hunan Province. Six duplicate tissue materials were collected from the root, stem, and leaf of *Kadsura coccinea* introduced from Huaihua, Hunan Province. After washing and drying with sterilized deionized water, they were put into a 2 mL cryopreservation tube. After quick freezing with liquid nitrogen, they were stored in a low-temperature refrigerator at −80°C for standby.

### 2.2. Method

#### 2.2.1. Metabolomic Analysis

Three biological replicates were taken from the root, stem, and leaf of *Kadsura coccinea* preserved at ultra-low temperature, and a total of 9 sample materials were analyzed for extensive targeted metabolome.

*(1) Sample Metabolite Extraction*. Nine samples of the root, stem, and leaf were taken, and the three of each were placed in a freeze dryer (Scientz-100F) for vacuum freeze-drying and then ground to powder with a grinder (MM 400 Retsch). Then, we weigh 100 mg of powder and dissolve it in 1.2 ml of 70% methanol extract. We vortex the extract for 30 s, hold the sample for 30 min, repeat the process 6 times, and place the vortex sample in the refrigerator at 4°C overnight. After centrifugation (rotating speed 12000 rpm, 10 min), we absorb the supernatant and use a microporous filter membrane (0.22 *μ*m). The samples were filtered and stored in the injection bottle for ultra-high-performance liquid chromatography-tandem mass spectrometry (UPLC-MS/MS) analysis.

*(2) UPLC-MS/MS Analysis*.

(1) Liquid phase conditions:
  1. Chromatographic column: Agilent SB-C18 1.8 *µ*m, 2.1 mm*∗*100 mm
  2. Mobile phase: phase A is ultrapure water (add 0.1% formic acid), and phase B is acetonitrile (add 0.1% formic acid)
  3. Elution gradient: the proportion of phase B is 5% in 0.00 min, the proportion of phase B increases linearly to 95% in 9.00 min and remains at 95% for 1 min, for 10.00–11.10 min, and the proportion of phase B decreases to 5% and balances with 5% to 14 min
  4. Flow rate: 0.35 ml/min; column temperature: 40°C; injection volume: 4 *μ*l
(2) Mass spectrum conditions:
Linear ion trap and three-pole quadrupole scanning were performed on a three-pole quadrupole linear ion trap mass spectrometer (Q TRAP), AB4500 Q TRAP UPLC/MS/MS system. The system is equipped with an electrospray ionization source ion spray interface, which can be controlled using Analyst 1.6.3 software (AB Sciex) to obtain two positive and negative ion modes. The operating parameters of the electrospray ion source are as follows: ion source, turbine spray; source temperature of 550°C; ion spray voltage of 5500 V (positive ion mode)/−4500 V (negative ion mode). The ion source gas I, ion source gas II, and curtain gas are set to 50, 60, and 25.0 psi, respectively, and the collision-induced ionization parameter is set to high value. In triple quadrupole and linear ion trap modes, 10 and 100 *μ*mol/L are used, respectively. The instrument tuning and mass calibration were carried out with mol/L polypropylene glycol solution. Triple quadrupole scanning was performed using multireaction monitoring mode and by setting the collision gas (nitrogen) to medium. By further optimizing the declustering voltage and collision energy, the declustering voltage and collision energy of each multireaction monitoring ion pair are obtained. According to the metabolites eluted in each period, a specific set of multireaction monitoring ion pairs are monitored in each period [9].

#### 2.2.2. Screening of Lignans-Related Components

After the samples from different parts of *Kadsura coccinea* were analyzed by UPLC-MS/MS, based on the self-built database of Baimike Technology Co. Ltd., the related components of lignans in *Kadsura coccinea* root, stem, and leaf were analyzed qualitatively and quantitatively according to the secondary spectrum information.

#### 2.2.3. Transcriptome Sequencing Analysis

*(1) Construction of Sequencing Library and Computer Sequencing*. The trizol method (Tiangen dp411 Kit) was used to extract the total RNA of 9 samples from the root, stem, and leaf of *Kadsura coccinea* for 3 biological repetitions. After passing the nucleic acid concentration and integrity test, the second-generation transcriptome sequencing library was constructed by using VAHTS Universal V6 RNA-seq Library Prep Kit for Illumina. It includes magnetic bead enrichment, mRNA reverse transcription to form cDNA, sequencing connector connection, PCR amplification, and enrichment of samples. After the library passed the quality inspection, the transcriptome of 9 samples was sequenced through Illumina NovaSeq 6000 platform.

*(2) Bioinformatics Analysis of Transcriptome Sequencing*. We filter the original data obtained, remove the joint sequence and low-quality reading sequence, and obtain high-quality reading sequence data. The data were assembled by Trinity software to obtain the UniGene Library of *Kadsura coccinea* root, stem, and leaf tissues. The quality of the sequencing library was evaluated by examining the randomness of mRNA fragmentation, the dispersion of the length of inserted fragments, the library capacity, and the adequacy of mapped reads compared to the UniGene library. The qualified database then carries out functional annotation and expression analysis on UniGene through BLAST and other software.

#### 2.2.4. Screening of Enzyme Genes Involved in Lignan Synthesis

By comparing and analyzing the transcriptome data of *Kadsura coccinea* root, stem, and leaf through various databases, we found the differentially expressed genes that are specifically expressed or highly expressed in different tissues and directly participate in lignan biosynthesis.

#### 2.2.5. “Transcription + Metabolism” Correlation Analysis of Lignan Biosynthesis Pathway

According to the results of differential metabolites and differential gene analysis of lignans, the differential genes and differential metabolites of the same group were mapped into the KEGG pathway map of lignans synthesis and, at the same time, KEGG enrichment analysis and correlation analysis between lignan differential genes and differential metabolites were carried out.

## 3. Results and Analysis

### 3.1. Metabonomic Analysis of *Kadsura coccinea*-Related Components

We take *Kadsura coccinea* root, stem, and leaf for 3 biological replicates, respectively, and a total of 9 sample materials for metabolomic analysis. Based on the UPLC-MS/MS detection platform and the database established by Baimaike Technology Co., Ltd., a total of 1000 metabolites were detected. These metabolites are divided into 13 categories, including 172 phenolic acids, 166 lipids, 134 flavonoids, 96 organic acids, 86 amino acids and their derivatives, 66 terpenes, 60 nucleotides and their derivatives, and 55 lignans and coumarins, as shown in Table 1. The contents of lignans, terpenoids, and alkaloids in roots were higher than those in stems and leaves. The content of flavonoids in roots was lower than that in stems and leaves.

### 3.2. Analysis of Lignans-Related Components

**Figure 1:**
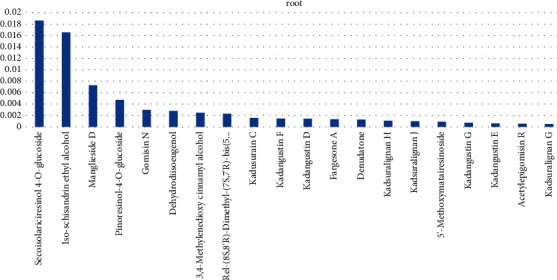
The relative content of lignans in the top 20 root samples of *Kadsura coccinea*.

Among the 55 primary metabolites of lignans and coumarins detected, there were 51 secondary metabolites of lignans. The lignans with high content in *Kadsura coccinea* roots are ring-opening isolarch phenol-4-O-glucoside, isoschisandrin B, mangliesin D, etc., as shown in Figure 1.

**Figure 2:**
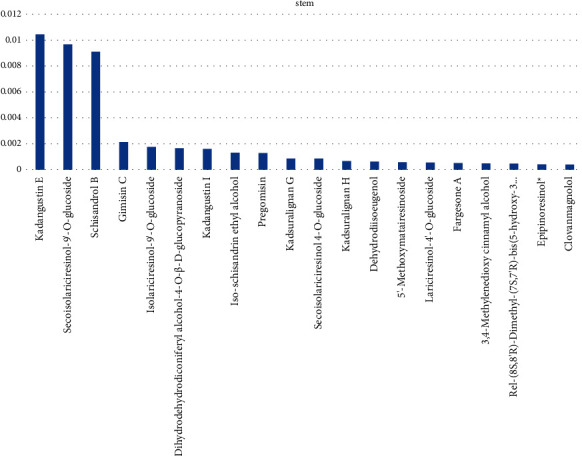
The relative content of lignans in the top 20 stem samples of *Kadsura coccinea*.

Lignans with high content in *Kadsura coccinea* stem include schisandrin E, ring-opening isolarch-9′- O-glucoside, schisandrin ethyl, etc., as shown in Figure 2.

**Figure 3:**
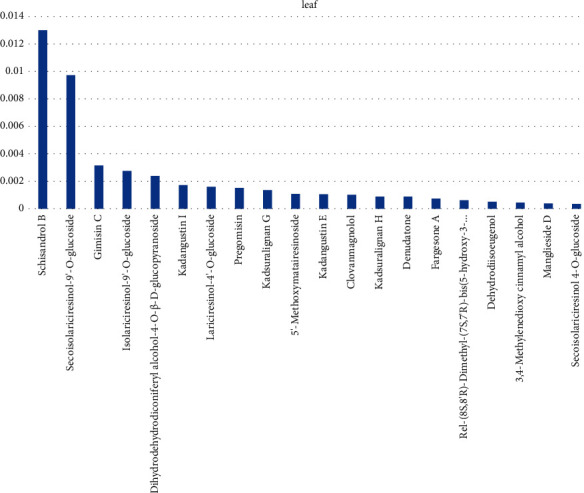
The relative content of lignans in the top 20 leaf samples of *Kadsura coccinea*.

The high content of lignans in *Kadsura coccinea* leaves includes schisandrin B, ring-opening isolarch-9′- O-glucoside, inulin C, etc., as shown in Figure 3.

### 3.3. Transcriptome Sequencing Results

Transcriptome sequencing was performed on 9 samples from *Kadsura coccinea* root, stem, and leaf. A total of 60.44 Gb Clean Data was obtained. The Clean Data of each sample reached 5.73 Gb, and the percentage of Q30 base was 94.35% or more. After assembly, a total of 54309 Unigenes were obtained. Among them, there are 16141 Unigenes with a length of more than 1 kb. The functional annotation of Unigenes includes the comparison with cog, go, KEGG, KOG, Pfam, Swiss-Prot, TrEMBL, eggnNOG, and NR databases, as shown in Table 2. A total of 26658 Unigene annotation results are obtained, of which 13683 Unigenes with a length of more than 1000 bases are annotated. Among all databases, the number of Unigenes annotated in the nonredundant protein (NR) database is the largest, reaching 25852, 12302 for 300∼1000 and 13550 for more than 1000, as shown in Table 2.

### 3.4. Gene Analysis of Enzymes Involved in Lignans Synthesis

Combined with the research on lignan biosynthesis of other species, we continued to excavate the enzyme genes related to lignan biosynthesis from the 26658 Unigenes annotated. Referring to the phenylpropane metabolic pathway (ko00940) and some other reported lignan biosynthesis pathways (ko00998) [10–15], a total of 13 classes of 137 Unigenes related to the lignan biosynthesis pathway were retrieved, of which the gene family with a large number is hydroxycinnamoyl transferase (HCT) polymerization protease (DIR) and cinnamoyl CoA reductase (CCR), 37, 21, and 16. Cinnamic acid-4-hydroxylase (C4H), coumaric acid-3-hydroxylase (C3H), and phenylalanine ammonia-lyase (PAL), respectively, have a small number of gene families, as shown in Table 3.

**Figure 4:**
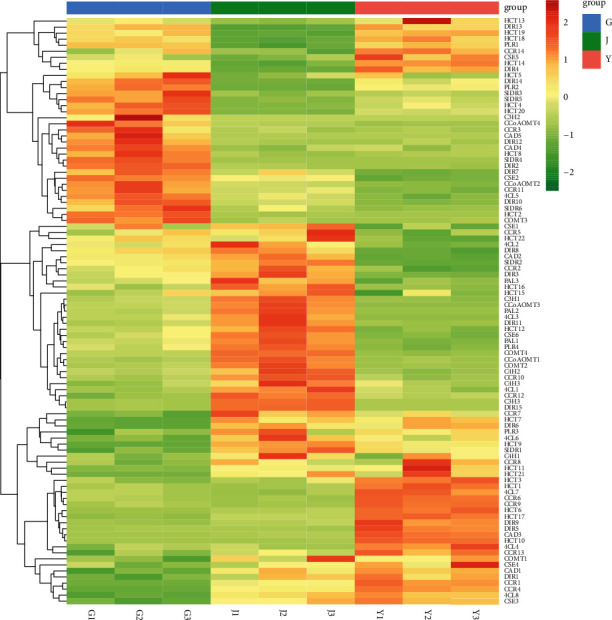
The relative expression of lignan synthase gene in *Kadsura coccinea* roots, stems, and leaves.

Furthermore, we analyze the relative gene expression of the 137 Unigenes related to lignan synthesis in 9 transcriptome samples of *Kadsura coccinea* root, stem, and leaf (3 replicates in each part) and cluster-analyze the differential genes, as shown in Figure 4. Heat map analysis showed that the total expression and species of *Kadsura coccinea* lignan synthase gene in stems and leaves were closer to those in roots. Caffeioyl COA oxymethyltransferase (CCoAOMT), coumaric acid-3-hydroxylase (C3H), and isolarch dehydrogenase (SIDR) are among several enzyme gene families, which are mainly expressed in roots and stems.

### 3.5. “Transcription + Metabolism” Correlation Analysis of Lignan Biosynthesis Pathway

Among the 137 Unigenes related to lignan synthesis obtained from the transcriptome, 30 Unigenes with high expression were screened. The correlation analysis was carried out in the phenylpropane metabolic pathway (ko00940) with the three lignans with the highest content in roots, stems, and leaves obtained from the metabolome analysis.

**Figure 5:**
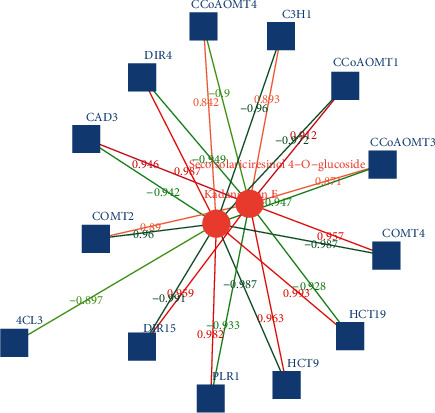
Correlation network of differentially expressed genes and metabolites in *Kadsura coccinea* lignan biosynthesis (root and stem).

In the process of root and stem comparison, 13 differential gene expressions were related to ring-opening isolarch phenol-4-o-glucoside and schisandrin E. Among them, there are 8 positive correlations and 4 negative correlations involving ring-opening isolarch phenol-4-o-glucoside. Among the positive correlations, hct9, dir15, comt4, and cad3 have relatively large correlations, as shown in Figure 5.

**Figure 6:**
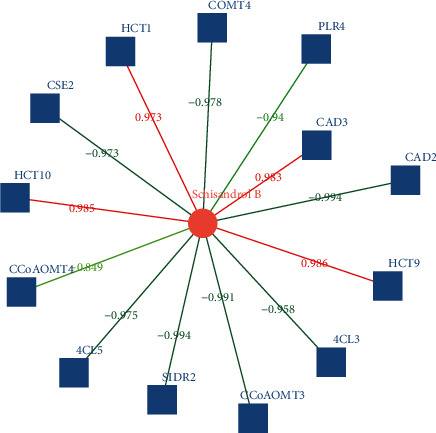
Correlation network of differentially expressed genes and metabolites in *Kadsura coccinea* lignan biosynthesis (root and leaf).

During the comparison of root and leaf, 13 differential gene expressions were related to schisandrin B, including 4 positive and 9 negative correlations. The four positive correlation genes were HCT9, HCT10, CAD3, and HCT1, as shown in Figure 6.

**Figure 7:**
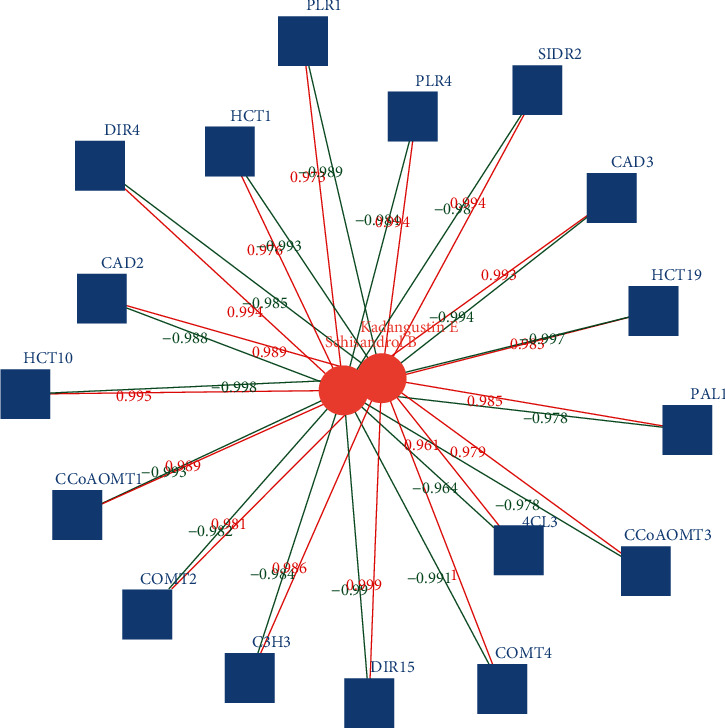
Correlation network of differentially expressed genes and metabolites in *Kadsura coccinea* lignan biosynthesis (stem and leaf).

In the comparison of stem and leaf, 17 differential gene expressions were related to schisandrin E and schisandrin B. Schisandrin E has 11 positive correlations and 6 negative correlations. In the positive correlations, COMT4, DIR15, PLR4, and SIDR2 are more correlated; Schisandrin B has 6 positive correlations and 11 negative correlations. Among the positive correlations, HCT10, DIR4, and CAD3 genes have relatively large correlations, as shown in Figure 7.

## 4. Discussion

Lignans are important secondary metabolites of plants, which play an important role in insect resistance and stress growth of plants. At the same time, lignans also have important pharmacological activities such as antitumor, anti-HIV, anti-inflammatory, liver protection, and antioxidation [16]. The research on chemical constituents of *Kadsura coccinea* has made rapid progress, but it mainly focuses on chemical composition analysis and pharmacological activity research. At present, the research on the lignan biosynthesis pathway of *Kadsura coccinea* has not been reported. In the research field of lignan biosynthesis in *Kadsura coccinea*, our research team used the method of metabolome combined with transcriptome analysis for the first time.

At present, the metabolomic analysis of *Kadsura coccinea* focuses on the study of relevant components of fruits and seeds or the study of flavonoids in different parts [17]. However, the metabolomic study of lignans, the main pharmacodynamic component of *Kadsura coccinea*, has not been carried out.

However, lignans and their derivatives are one of the main chemical constituents isolated from black tigers. Lu et al. [18] used UV-vis spectrophotometry to determine the content of total lignan in black tigers from three different producing areas in Guangxi Province and found that the content of total lignan in black tigers from different producing areas was different, with the content between 0.93% and 2.11%. The main lignan compounds isolated from *Schisandra chinensis* are biphenyl cyclooctene lignans [19]. At present, there are 49 biphenyl cyclooctadiene lignans isolated and identified from black tiger, which can be divided into three categories: the common biphenyl cyclooctadiene type, spirobenzofurane biphenyl cyclooctadiene type, and 6,9 oxbridge biphenyl cyclooctadiene type lignans [20]. Through ultra-high-performance liquid chromatography-tandem mass spectrometry, the research team analyzed the related components of lignans in the root, stem, and leaf of *Kadsura coccinea* and obtained a total of 51 lignans, and the content in the root is higher than that in the stem and leaf. The high content of lignans in roots, stems, and leaves includes ring-opening isolarch phenol-4-o-glucoside, narrow leaf schisandrin E, and schisandrin B. This study enriches the study of lignans in different parts of *Kadsura coccinea* and lays a solid foundation for the further development of lignans in different parts of *Kadsura coccinea*.

At present, there is no report on the study of enzyme genes related to lignan biosynthesis in *Kadsura coccinea*. After obtaining UniGene data information through transcriptome sequencing, according to the reported enzymes involved in lignan biosynthesis and metabolism in other plants, this study further searched the information related to lignan synthesis in *Kadsura coccinea* and a total of 13 classes of 137 Unigenes related to lignan biosynthesis pathway were retrieved. Among them, CAD, C4H, CCoAOMT, 4CL, CCR, C3H, PAL, HCT, COMT, and CSE belong to the upstream stage of lignan biosynthesis, which is responsible for the production of lignan precursor coniferol from phenylalanine through a series of reactions. DIR, PLR, and SIDR are lignans such as ring-opening isolarch alcohol and Mohanone, which are further generated from coniferol through turpentine alcohol and larch alcohol. The analysis of differential genes in different parts showed that the overall expression and species of *Kadsura coccinea* lignan synthase gene in stems and leaves were closer than those in roots. CCoAOMT, C3H, and SIDR gene families are mainly expressed in roots and stems. Furthermore, we analyze the transcriptional groups of 11 genes related to HCRD and PLT. In this study, the gene information of the *Kadsura coccinea* lignan biosynthesis enzyme was obtained for the first time, which laid a good foundation for the cloning and molecular breeding of key enzyme genes of *Kadsura coccinea* lignan biosynthesis.

## 5. Conclusion

The *Kadsura coccinea* has great economic value, and it has become a characteristic industrial source in Hunan, Fujian, and other provinces. However, due to the lack of research and development on black tiger, there are not many kinds of black tiger-related products with low added value and industrial development is restricted to a certain extent. Although we have a comprehensive understanding of the chemical composition of the black tiger, the study of its efficacy and the basis of its pharmacological substances is still very weak. Firstly, many chemical components still lack activity and mechanism research. Secondly, pharmacological studies are not comprehensive, in-depth, and systematic, which cannot provide sufficient support for the clinical application and efficacy development of the black tiger. Thirdly, the quantitative study of its chemical composition is obviously lacking, which leads to the great limitation of the standard study. Finally, there is a lack of metabolism and metabolite activity in in vivo research. These problems need to be focused on and solved in future research work.

## Figures and Tables

**Table 1:** The average value of species, quantity, and relative content of metabolites in roots, stems, and leaves of *Kadsura coccinea*.

Primary metabolites	Number	Relative content		
		Root	Stem	Leaf
Lignans and coumarins	55	7.57*E*-02	4.85*E*-02	4.74*E*-02
Terpenoids	66	4.95*E*-02	2.80*E*-02	3.74*E*-02
Flavonoids	134	5.18*E*-02	1.32*E*-01	2.47*E*-01
Phenolic acids	172	1.56*E*-01	2.14*E*-01	2.21*E*-01
Lipid	166	1.23*E*-01	1.22*E*-01	1.24*E*-01
Organic acid	134	5.31*E*-02	7.11*E*-02	4.63*E*-02
Amino acids and their derivatives	86	2.17*E*-01	1.63*E*-01	8.52*E*-02
Nucleotides and their derivatives	60	1.41*E*-02	1.82*E*-02	2.20*E*-02
Alkaloid	26	1.35*E*-01	5.66*E*-02	2.78*E*-02
Tannin	12	2.61*E*-02	1.30*E*-02	3.84*E*-03
Other classes	127	9.85*E*-02	1.34*E*-01	1.38*E*-01

**Table 2:** The number of single gene clusters annotated in different databases.

Functional database	Number of single gene clusters annotated	300 ≤ single gene cluster length < 1000	Single gene cluster length ≥ 1000
COG_Annotation	6,661	2,206	4,455
GO_Annotation	20,579	9,162	11,414
KEGG_Annotation	16,434	6,820	9,614
KOG_Annotation	13,605	5,614	7,991
Pfam_Annotation	17,561	6,625	10,936
Swissprot_Annotation	15,424	5,871	9,553
TrEMBL_Annotation	25,161	11,595	13,566
eggNOG_Annotation	21,083	9,124	11,959
NR_Annotation	25,852	12,302	13,550
All_Annotated	26,658	12,972	13,683

**Table 3:** The major lignan synthase genes in *Kadsura coccinea*.

Gene family	Number of Unigenes	Code ID
Cinnamyl alcohol dehydrogenase (CAD)	11	c138243, c127253, c100870, c100870, c132969, c143622, c75605, c139976, c142940, c106854, c139288
Cinnamate-4-hydroxylase (C4H)	3	c133158, c141289, c140162
Caffeioyl COA oxymethyltransferase (CCoAOMT)	5	c123906, c147567, c130267, c134322, c139948
4-Coumarinyl CoA ligase (4CL)	11	c141218, c148238, c148167, c139095, c105572, c147311, c152599, c147707, c148297, c141218, c142717
Cinnamoyl CoA reductase (CCR)	16	c141261, c135703, c120215, c140397, c141246, c146949, c136448, c140375, c138246, c141087, c134396, c147312, c144833, c149012, c140534, c129111
Coumaric acid-3-hydroxylase (C3H)	3	c138815, c130179, c145761
Phenylalanine ammonia-lyase (PAL)	3	c145137, c142300, c125745
Hydroxycinnamoyl transferase (HCT)	37	c148893, c149025, c143322, c137863, c135415, c132343, c138259, c137078, c130160, c135415, c145853, c132343, c150077, c139674, c136799, c149948, c12859, c134687, c133462, c146001, c136937, c144136, c136395, c146817, c149992, c140186, c142265, c145173, c131394, c136937, c93393, c148160, c125532, c135415, c132085, c135897, c146817
Caffeic acid oxymethyltransferase (COMT)	6	c146701, c106776, c122011, c140134, c144220, c149283,
Isolaricin dehydrogenase (SIDR)	10	c127415, c145168, c114390, c145090, c135982, c128786, c130422, c132787, c142699, c126873
Polymerized protease	21	c131826, c182448, c122470, c135239, c131102, c145124, c131090, c134134, c134002, c114144, c134644, c135894, c121895, c126128, c132052, c135894, c100228, c125953, c112255, c133684, c144151
Terpineol reductase	4	c138332, c140550, c145566, c147861
Caffeioyl shikimate esterase (CSE)	7	c139639, c148145, c142263, c143532, c141052, c139096, c134673

## Data Availability

The data used to support the findings of this study are available from the corresponding author upon request.
